# Psychiatric antecedents in young patients with first episode psychosis: what relevance for clinical outcomes?

**DOI:** 10.1007/s00406-025-01981-6

**Published:** 2025-02-28

**Authors:** Lorenzo Pelizza, Fabio Catalano, Emanuela Leuci, Emanuela Quattrone, Derna Palmisano, Simona Pupo, Giuseppina Paulillo, Clara Pellegrini, Pietro Pellegrini, Marco Menchetti

**Affiliations:** 1 Department of Biomedical and Neuromotor Sciences, Alma Mater Studiorum-Università di Bologna, c/o Istituto di Psichiatria “Paolo Ottonello”-via Pepoli, 5, 40126 Bologna, BO Italy; 2 Department of Mental Health and Pathological Addiction, Azienda USL di Parma, Parma, PR Italy; 3 Pain Therapy Service, Department of Medicine and Surgery, Azienda Ospedaliero-Universitaria di Parma, Parma, PR Italy

**Keywords:** First episode psychosis, Antecedents, Early psychosis, Early Intervention in Psychosis, Outcome, Help-seeking

## Abstract

Examining psychiatric antecedents and help-seeking behavior for people with First Episode Psychosis (FEP) could help understand determinants for timely care pathways, decrease the “Duration of Untreated Psychosis” (DUP), and consequently improve their prognosis. The aims of this study were: (1) to calculate the proportion of FEP participants with previous contact with mental healthcare services recruited within a specialized “Early Intervention in Psychosis” service, and (2) to longitudinally compare sociodemographic, clinical, and treatment parameters between FEP patients with and without psychiatric antecedents across a 2-year follow-up period. All participants (aged 12–35 years) were enrolled within the “Parma Early Psychosis” (Pr-EP) program. At baseline, they completed the Health of the Nation Outcome Scale (HoNOS). A mixed-design ANOVA and a Kaplan–Meier survival analysis were used. Of the 489 FEP participants, 204 (41.7%) patients had prior contact with mental health services. In 83% of cases, a care discontinuity was observed. Main psychiatric antecedents at entry were personality disorders (32.8%), anxious-depressive disorder (28.9%), conduct disorder (16.2%), and learning disorder (9.8%). FEP subjects with antecedents were more likely to receive a diagnosis of schizophrenia at baseline. Having previous contact with psychiatric services resulted to be a predictor of poorer clinical and functional outcome. It is very important to carefully monitor mental health suffering and related help-seeking-behavior in young patients typically manifested in their early 20s, especially in terms of psychosis prevention. Particular attention should also be given to service engagement as care continuity within adolescent-adult transition.

**Supplementary Information:**

The online version contains supplementary material available at 10.1007/s00406-025-01981-6.

## Introduction

The *development of psychopathology* in youth has been examined as potential prodrome for the onset of psychosis [1]. Cognitive disturbances, social isolation, anxiety, and depression are frequently reported before a First Episode Psychosis (FEP), even many years before the onset of the disorder [2]. In many cases, psychological distress and *help-seeking behavior* for disorders other than psychosis may be considered as crucial clinical determinants for timely care pathways in FEP patients and for decreasing the *Duration of Untreated Psychosis* (DUP) [3]. In this respect, the DUP has been hypothesized to be “neurotoxic” [4] and longer DUP has been significantly related to poorer outcome (social, clinical, and global) in individuals with FEP [5].

In the last two decades, considerable efforts have been made to detect (and treat) the early phases of psychosis, especially focusing on subjects with at-risk mental states and *attenuated psychotic psychopathology* [6]. These strategies mainly aim to identify prodromal symptoms of psychosis as early as possible in order to reduce the time interval between the onset of a psychotic disorder, its diagnosis and the start of treatment [7]. However, some authors observed that mental health clinicians do not seem to timely identify the development of psychosis when treating other disorders [8], or perhaps they consider the psychotic features as secondary to other problems [9]. If a certain proportion of subjects who are likely to develop psychosis in the future do seek help in specialized psychiatric services (including child and adolescent mental healthcare centers), screening for attenuated psychotic experiences might be an effective strategy to prevent a lengthy period of untreated psychosis and to prevent/postpone a FEP [10]. Indeed, at the first contact, they often manifest unspecified anxious-depressive conditions and/or poorly defined clinical problems (such as anomalous self-experiences, basic symptoms, soft neurological signs, negative and cognitive symptoms) that significantly affect school functioning and social integration with peers [11]. In this respect, the recent paradigm shift from “Early Intervention in Psychosis” (EIP) services to “Youth Mental Health” (YMH) services sought to overcome these potential diagnostic delay factors and to promote timely identification of FEP [12]. Indeed, in recent years, there has been a global push for youth-focused services under the aegis of the World Health Organization [13], and YMH service reform has been gaining ground, first in Australia, then in Europe and worldwide [14]. Specifically, in Australia, the “Fourth National Mental Health Plan”, as well as prioritizing the expansion of accessible YMH services as one of its action areas, recommended the development of constitutive principles to guide the establishment of youth-focused services [15]. Among them, those currently considered the most important are: to acknowledge and incorporate the full continuum of service response; to employ evidence-informed practice; to ensure smooth pathways and ease of access into services; to embody a “youth-friendly” ethos; to facilitate youth empowerment, agency and self-determination; to take into account the developmental stage of the young person; to prioritize youth “at-risk” of, or experiencing, severe mental ill-health; to collaborate with other services in the treatment system; to provide family-sensitive practices; and to take an integrated, holistic approach with a recovery focus [16]. However, very few investigations examined help-seeking behavior before and during the *prodromal stage of psychosis* [17]. In this respect, the concept of “prodromal phase” is usually used in retrospective research to understand the antecedents of patients that have developed a specific disorder or episode. Indeed, according to Ortiz-Orendain and colleagues [18], a prodrome may be correctly defined as “the period of disturbance which represents a deviation from a person’s previous experience/behavior prior to the development of the florid features of a disorder”. Thus, in FEP research, the initial prodrome should entail the clinical antecedents that occur prior to the FEP. Specifically, previous retrospective research reported an 80% prevalence of prodromal disorders in FEP patients [19] and relevant proportions of FEP individuals that sought help for an Axis I or II diagnosis prior to the psychosis onset (ranging between 40 and 80%) [20]. Furthermore, in a more recent US population-based registry study, approximately 76% of participants with first episode schizophrenia had initial prodromes [18]. Most of them (80%) started seeking help before the age of eighteen (mean age at first visit for mental health problems = 12 years) and were more likely to have antecedent diagnoses of neurodevelopmental disorders such as ADHD, conduct disorders, and intellectual disabilities.

In a Dutch adult population, the largest groups of FEP subjects with previous specialist contact were referred for treatment for substance use disorders, mood and anxiety disorders, and adjustment disorders [21]. Specifically, whereas women had more affective, anxiety and adjustment disorders in their help-seeking history, men had been treated more often for substance abuse problems. The mean time from first contact to first diagnosis of psychosis was about 87 months. In this respect, the authors concluded that people first diagnosed with substance use, anxiety and mood disorders developed psychosis sooner than people with no antecedent diagnoses at the first contact, also suggesting that detection and interventions in both secondary and primary care services would be helpful to reduce the DUP. However, they had no knowledge about treatment history of patients who previously had specialist contact with child and adolescent psychiatric services.

Overall, these findings and proportions of help-seeking behavior are based on small sample sizes. A more accurate estimate of the prevalence of these psychiatric antecedents and requests for specialist help in larger clinical populations entering specialized “Early Intervention in Psychosis” (EIP) services before the onset of psychosis is thus needed. The *aims* of the current investigation were: (1) to calculate what proportion of FEP people treated within an EIP program previously had been help-seeking in mental healthcare services at the prodromal stage, and (2) to longitudinally compare sociodemographic, clinical, and treatment parameters between FEP patients with and without psychiatric antecedents across a 2-year follow-up study. This study may also usefully contribute to informing mental health professionals working in YMH services on the main psychiatric antecedents that can (often non-specifically) characterize the psychopathological trajectories of psychosis, promoting timely identification of prodromal states.

## Methods

### Setting and participants

All participants were consecutively enrolled within the “Parma Early Psychosis” (Pr-EP) protocol between January 2013 and December 2021. Specifically, the reason for stopping the enrollment process in 2021 was related to the time of the deadline for recruitment according to the study protocol approved by the local ethics committee. The Pr-EP program is a specialized EIP program implemented across all adolescent and adult mental healthcare services in the Parma Department of Mental Health (Northern Italy) [22]. The catchment area included approximately 500.000 inhabitants. The treated incidence of mental disorders in 2021 was approximately 70 cases per 100.000 inhabitants, with an annual treated incidence of FEP ranging between 11 and 15 cases per 100 000 inhabitants across our recruitment time [23].

*Inclusion criteria* were: (a) specialist help-seeking request, (b) enrollment in the Pr-EP program, (c) age 12–35 years, (d) presence of FEP within one of the following DSM-5 diagnoses: schizophrenia, bipolar disorder or major depressive disorder with psychotic features, delusional disorder, brief psychotic disorder, schizophreniform disorder, and psychotic disorder not otherwise specified [24], and (e) “Duration of Untreated Psychosis” (DUP) of < 2 years. The DUP was defined as the time interval (in months) between the onset of overt psychotic features and the first antipsychotic prescription [25]. Its length was selected in accordance to the usual time limit to provide effective interventions within the EIP paradigm [26]. For clarity, the general initial criteria for inclusion in the Pr-EP program also considered a wider age range (12–54 years) for FEP patients and adolescents/young adults (aged 12–25 years) at Clinical High Risk for Psychosis (CHR-P) (see [22] for details).

*Exclusion criteria* were: (a) history of previous psychotic episodes (i.e., outside the current illness episode), (b) known intellectual disability (IQ < 70), and (c) neurological or other medical disease with psychiatric symptoms. Past exposure to antipsychotic medication (i.e., at any dosage and in previous illness episode before the Pr-EP enrollment) was considered as a “functional equivalent” of past psychotic episode [27]. Indeed, the original EIP paradigm psychometrically defined the “psychosis threshold” as essentially that at which antipsychotic medication would probably be started in the common clinical practice [28]. All participants (including minors and their parents) provided their written informed consent for study participation. The research received local ethical approvals (AVEN Ethics Committee protocol n. 559/2020/OSS*/AUSLPR) and adhered to the Declaration of Helsinki (and its later amendments). The data are not publicly available due to privacy/ethical restrictions.

### Assessment

The presence of FEP at entry was detected using the psychometric criteria of the “Comprehensive Assessment of At-Risk Mental States” (CAARMS), approved Italian version [29]. Moreover, the baseline DSM-5 diagnosis was formulated by a minimum of two trained PARMS team members using the Structured Clinical Interview for DSM-5 mental disorders (SCID-5) [30].

The clinical and outcome assessment was based on the Health of the Nation Outcome Scale (HoNOS) [26], a widely used clinical instrument for evaluating mental health and social functioning in severe mental illness patients, including young people with FEP [31]. We assessed four main outcome domains as suggested by Morosini and his team [32]: “Psychiatric Symptoms”, “Impairment”, “Behavioral Problems”, and “Social Problems”. This assessment was conducted both at baseline and every 12 months during the 2-year follow-up period by trained PARMS team members. Its approved Italian version maintained a good to excellent interrater reliability through supervision sessions and scoring workshops [33].

Finally, a sociodemographic/clinical chart was completed at presentation, capturing a broad range of parameters including gender, age at entry, years of education, employment and migrant status, past specialist contact and previous psychiatric antecedents, current substance abuse, DUP, and pharmacological therapy.

### Procedures

After CAARMS and SCID-5 interviews, FEP participants were divided in FEP + and FEP– subgroups depending on having or not a past specialist contact for mental problems. These two subsamples were enrolled in the Pr-EP program and were tested for clinical outcomes with the HoNOS both at baseline and along the 2-year follow-up period. Moreover, 2-year incidence rates of service disengagement, new hospitalization, and new suicide attempt were also calculated.

The Pr-EP program provided a 2-year comprehensive treatment package including a psychopharmacological therapy and a multi-component psychosocial intervention (combining individual psychotherapy based on cognitive-behavioral approach, psychoeducational sessions for family members and a recovery-oriented case management) in accordance with the current EIP guidelines [34–36].

### Statistical analysis

Collected data were analyzed using the Statistical Package for Social Science (SPSS) 15.0 for Windows [37]. All tests were two-tailed, with a significance level set at 0.05. There were no missing data. In between-group comparisons, the Chi-squared (Χ^2^) test for categorical variables and the Mann–Whitney U test for continuous measures were used. All p values were corrected for multiple comparisons. As for outcome parameters that had as endpoint the time when a specific event occurs, we performed Kaplan–Meier survival analyses to consider the different duration of follow-ups and participants who dropped out from the study protocol. Finally, a mixed-design ANOVA analysis was performed to evaluate the temporal stability of HoNOS scores within and between the two FEP + and FEP- subgroups across the 2-year follow-up period.

## Results

**Fig. 1:**
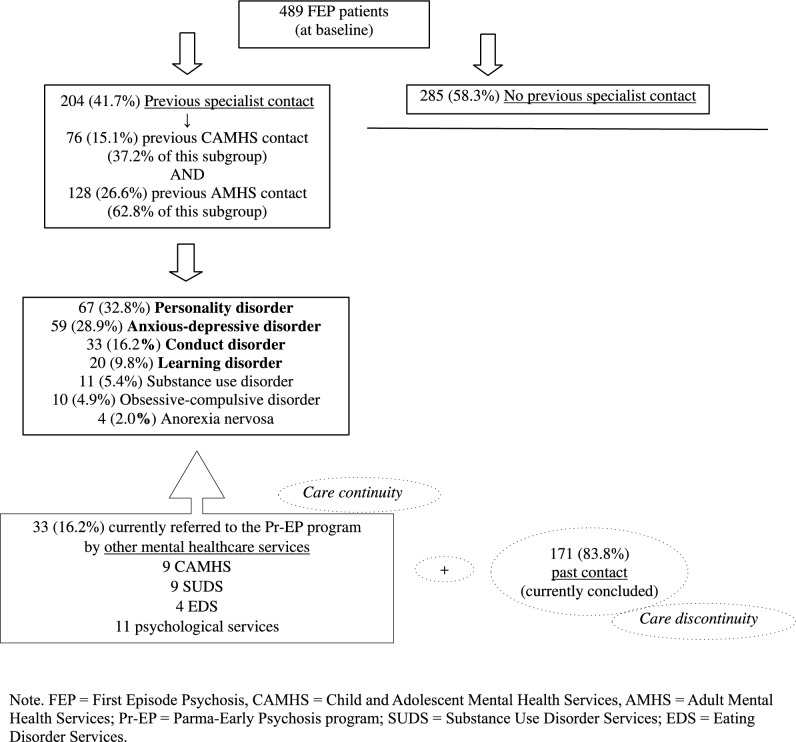
Prevalence rate of FEP patients with and without previous specialist contact. *FEP* First Episode Psychosis, *CAMHS* Child and Adolescent Mental Health Services, *AMHS* Adult Mental Health Services, *Pr-EP* Parma-Early Psychosis program, *SUDS* Substance Use Disorder Services, *EDS* Eating Disorder Services

A total of 489 FEP patients were enrolled in this investigation: 204 (41.7%) had a previous specialist contact and were included in the FEP + subgroup, while 285 were grouped in the FEP- subsample (Fig. 1). Age at entry for FEP + individuals was 25.40 ± 6.23 years, while age at the first contact was 21.44 ± 7.82 years. The main DSM-5 diagnoses at the earlier contact were: personality disorder (32.8%), anxious-depressive disorder (28.9%), conduct disorder (16.2%), and learning disorder (9.8%). Finally, 76 (37.2%) participants of the FEP + total subsample had a previous specialist contact within Child/Adolescent Mental Health Services (CAMHS), while 128 (62.8%) within Adult Mental Health Services (AMHS). Specifically, only 33 (16.2% of the FEP + total subgroup) individuals were directly referred to the Pr-EP program by other mental healthcare services within a clinical pathway of care continuity, while in the remaining 171 (83.3%) subjects their past specialist contact ended with their care retention in mental health services being terminated (care discontinuity).

(Fig. 1 here).

Sociodemographic and clinical comparisons between the two FEP subgroups are summarized in the Table 1. At baseline, FEP patients with previous specialist contact were more likely to be single and to have longer DUP and higher prevalence rate of DSM-5 schizophrenia diagnosis, while FEP- individuals were more frequently referred by the emergency room and were more likely to have higher benzodiazepine prescription rate. No statistically significant inter-group differences were found in terms of clinical outcomes (i.e., service disengagement, new hospital admission, and new suicide attempt) across the 2 years of follow-up (see Table 1 and Supplementary Materials [Table S1 and Figure S1] for details).

**Table 1:** Sociodemographic and clinical comparisons in the two FEP subgroups

Variable	FEP+ (n = 204)	FEP− (n = 285)	X^2^/z	p
Gender (males)	126 (61.8%)	179 (62.8%)	0.055	0.815
Ethnic group (white Caucasian)	174 (85.3%)	232 (81.4%)	1.277	0.258
Migrant Status	28 (13.7%)	52 (18.2%)	1.775	0.183
Age (at entry)	25.51 ± 6.65	25.32 ± 5.99	− 0.356	0.722
Education (in years)	11.30 ± 2.74	11.62 ± 2.93	1.533	0.125
Civil status (single)	157 (77.4%)	182 (63.9%)	9.596	**0.002**
Living status (living with parents)	170 (83.3%)	223 (78.2%)	2.040	0.361
NEET	109 (53.4%)	150 (52.6%)	0.965	0.617
Source of referral				
Primary care	73 (35.8%)	86 (30.2%)	0.089	0.902
Family members	23 (11.3%)	26 (9.1%)	1.231	0.286
Self-referral	18 (8.8%)	18 (6.3%)	1.245	0.277
Emergency room	49 (24.0%)	105 (36.8%)	4.956	**0.038**
School/social services	8 (3.9%)	17 (6.0%)	1.677	0.115
Other health services	33 (16.2%)	33 (11.6%)	1.774	0.108
DUP (in months)	12.10 ± 10.91	8.28 ± 8.85	− 4.645	**0.0001**
Previous hospitalization	89 (43.6%)	124 (43.5%)	0.001	0.979
Previous suicide attempts	8 (3.9%)	18 (6.3%)	1.354	0.245
Substance misuse at entry	74 (36.3%)	114 (40.0%)	0.697	0.404
Baseline AP prescription	176 (86.3%)	241 (84.6%)	0.278	0.598
Equivalent dose of risperidone (mg/day)	2.95 ± 2.74	2.97 ± 2.57	− 0.357	0.721
Baseline AD prescription	32 (15.7%)	57 (20.0%)	1.486	0.223
Baseline MS prescription	29 (14.2%)	37 (13.0%)	0.155	0.694
Baseline BDZ prescription	58 (28.4%)	110 (38.6%)	5.447	**0.020**
Baseline acceptance of individual psychotherapy proposals	155 (76.0%)	217 (76.1%)	0.002	0.967
Baseline acceptance of family psychoeducation	129 (63.2%)	165 (57.9%)	1.414	0.234
Baseline acceptance of case management	161 (78.9%)	206 (72.3%)	2.800	0.094
Baseline DSM-5 diagnosis				
Schizophrenia	116 (56.9%)	136 (47.7%)	3.980	**0.046**
Affective psychosis	51 (25.0%)	89 (31.2%)	1.873	0.245
Brief psychotic disorder	25 (12.3%)	44 (15.4%)	1.670	0.342
Psychotic disorder NOS	10 (4.9%)	11 (3.9%)	1.332	0.875
Schizophreniform disorder	2 (1.0%)	5 (1.8%)	0.090	0.921
Baseline HoNOS scores				
Behavioral problems	3.61 ± 2.50	3.94 ± 2.44	− 1.584	0.113
Impairment	3.08 ± 2.02	3.28 ± 2.18	− 0.688	0.492
Psychiatric symptoms	10.42 ± 2.99	9.98 ± 3.55	− 1.132	0.258
Social problems	7.62 ± 3.73	7.76 ± 4.01	− 0.297	0.767
Total score	24.73 ± 7.85	24.95 ± 9.16	− 0.171	0.864
2-year drop-out incidence rate	47 (23.0%)	80 (28.1%)	1.565	0.211
2-year new hospitalization incidence rate	31 (24.2%)	63 (22.1%)	0.224	0.704
2-year new suicide attempt incidence rate	12 (5.9%)	9 (3.2%)	2.147	0.143
2-year functional recovery incidence rate	143 (70.1%)	202 (70.9%)	0.035	0.852
2-year HoNOS functional remission	32 (41.2%)	165 (40.0%)	0.124	0.725

Frequencies (and percentages) and mean ± standard deviation are reported. Chi-square (X^2^) and Mann–Whitney U (z) test values are reported. Statistically significant p values are in bold

*FEP* First Episode Psychosis, *FEP+* FEP patients with previous specialist contact, *FEP−* FEP patients without previous specialist contact, *NEET* Not [engaged] in Education, Employment or Training, *DUP* Duration of Untreated Psychosis, *AP* Antipsychotic medication, *LAI-AP* Long-Acting Injection Antipsychotic medication, *AD* Antidepressant medication, *MS* Mood Stabilizer, *BDZ* Benzodiazepine, *DSM-5* Diagnostic and Statistical Manual of mental disorders—5th Edition, *HoNOS* Health of the Nation Outcome Scale, *Functional recovery* return to school/work, *HoNOS functional remission* HoNOS item 9, 10 and 11 subscores < 2

(Table 1 here).

**Fig. 2:**
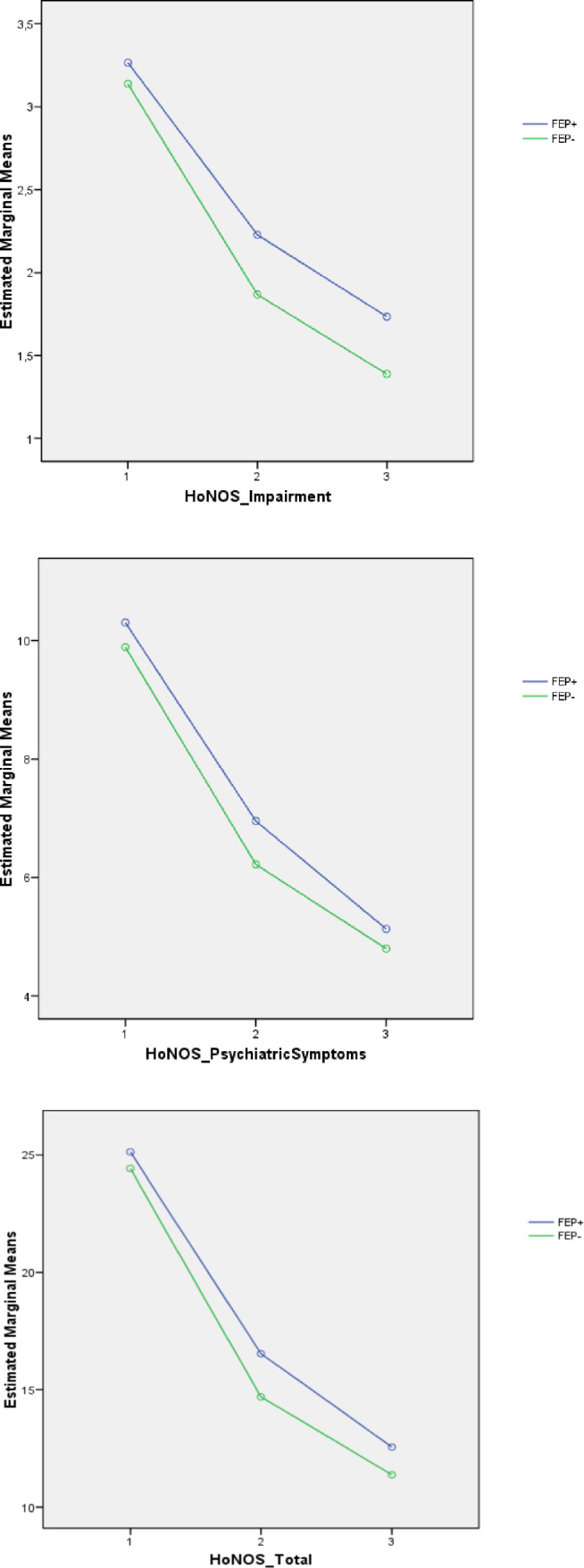
Mixed design ANOVA results: profile plots of statistically relevant HoNOS scores across the 2-year follow-up period in the two FEP subgroups. *FEP* First Episode Psychosis, *FEP+* FEP patients with previous specialist contact, *FEP−* FEP patients without previous specialist contact, *HoNOS* Health of the Nation Outcome Scale

Mixed-design ANOVA analysis results showed a statistically significant effect of time on all HoNOS scores (Table 2) and a relevant statistical trend (p < 0.1) on group effect for HoNOS “Impairment” and “Psychiatric Symptoms” domain subscores, as well as for HoNOS total scores. Specifically, as indicated by estimated marginal means across the follow-up (Fig. 2), compared to FEP + , FEP- patients had lower scores in HoNOS “Impairment” and “Psychiatric Symptoms” domains, as well as lower HoNOS total scores.

**Table 2:** Mixed-design ANOVA results: psychopathological and outcome parameters across the 2-year follow-up period in the two FEP subgroups

Variable	Time effect				Group effect (FEP + vs. FEP-)				Interaction effect (time x group)			
	df	F	p	η^2^	df	F	p	η^2^	df	F	p	η^2^
HoNOS Behavioral problems	1.6	275.412	**0.0001**	0.429	1	.168	0.682	0.001	1.6	2.646	0.100	0.006
HoNOS Impairment	1.5	186.372	**0.0001**	0.337	1	3.214	**0.074**	0.009	1.5	2.019	0.318	0.003
HoNOS Psychiatric symptoms	1.7	450.818	**0.0001**	0.551	1	3.072	**0.080**	0.008	1.7	0.737	0.457	0.002
HoNOS Social problems	1.6	232.278	**0.0001**	0.388	1	1.674	0.196	0.005	1.6	0.058	0.913	0.001
HoNOS total score	1.6	561.052	**0.0001**	0.695	1	2.904	**0.087**	0.008	1.6	1.049	0.338	0.003

Variable (group effect)	FEP+				FEP-			
	EEM	SE	95% CI Lower upper		EEM	SE	95% CI Lower upper	
HoNOS impairment	2.409	0.177	2.179	2.640	2.131	0.102	1.931	2.331
HoNOS psychiatric symptoms	7.464	0.213	7.046	7.882	6.972	0.184	6.610	7.333
HoNOS total score	18.074	0.548	16.996	19.152	16.831	0.474	15.898	17.764

As all Mauchly’s tests of sphericity are statistically significant (p < 0.05), Greenhouse–Geisser corrected degrees of freedom to assess the significance of the corresponding F value are used. Statistically significant p values are in bold. Statistical trends in p value (p < 0.01) are underlined. Statistical trends for p value are in bold

*ANOVA* analysis of variance, *FEP+* First Episode Psychosis with previous specialist contact, *FEP−* First Episode Psychosis without previous specialist contact, *df* degrees of freedom, *F* F statistic value, *HoNOS* Health of the Nation Outcome Scale, *p* statistical significance, *η*^*2*^ partial eta squared, *T0* baseline assessment time, *T2* 2-year assessment time, *EMM* estimated marginal mean, *SE* standard error; *95% CI* 95% confidence intervals for EMM

(Table 2 and Fig. 2 here).

Kaplan–Meier survival analysis on service disengagement, new hospitalization and suicide attempt showed no statistical difference between the two subgroups as reported in Table S1.

(Table S1).

## Discussion

Examining psychiatric antecedents in young FEP people may be an interesting opportunity to better define clinical trajectories in both psychopathological and diagnostic/prognostic terms [20]. In this investigation, 41.7% of FEP participants had previous contact with mental health services before entering the Pr-EP program. Specifically, most of them showed prior psychiatric antecedents without a current retention in care within generalist/first-level mental healthcare centers (care discontinuity). These findings highlight a critical problem for clinical practice: i.e., a relevant part (more than a third) of young FEP individuals lack sufficient monitoring for effective psychosis prevention and for a potential detection of an at-risk mental state of psychosis within help-seeking behaviors in a crucial age range [38, 39]. In this respect, it is necessary to underline the crucial role of pathways to care in defining the length of help-seeking and the appropriate access to specialist care. Specifically, the recent paradigm shift from EIP to YMH services could overcome some potential diagnostic delay factors (e.g., misdiagnosis, diagnostic underestimation of attenuated psychotic symptoms, considering psychotic features as secondary to other problems) [9] and promote timely identification of FEP [12], so as to potentially reduced the DUP and improve long-term outcomes.

Furthermore, in a noteworthy percentage of cases (approximately 1/5) our care discontinuity interested the transition age from CAMHS to AMHS. In this respect, the pan-European survey by the MILESTONE Consortium on the architecture and functioning clearly indicated that the organization of services is generally not based on patients’ perspectives and expectations (as they should be), and that this distance from users’ needs is even more critical as it fails to match the natural course of emerging severe mental illnesses in young individuals [40]. Indeed, youths aged 12–25 years have the highest prevalence and incidence of severe mental disorders across the lifespan, while also having the worst service engagement with CAMHS and AMHS compared to all other age groups [41]. As this unfortunate division of mental health care along the pediatric/adult model (mainly due to the traditional organization of somatic medicine) cuts across the age when the risk for severe mental illnesses peaks (with obvious consequences in terms of discontinuity of care, under-treatment, and unmet needs) [42], we therefore cannot postpone a radical review of the architecture and resourcing of health care for young patients in transition from childhood to adulthood any longer [28, 43]. However, our baseline prevalence of past specialist contact is lower than those reported in comparable, international epidemiological studies, ranging from 56 to 75% [18–21]. This difference in prevalence may be due to differences in the mean age at presentation. Indeed, our FEP population was older compared to samples recruited in previous investigations (25.40 Vs 20.40 years). However, overall considered, these findings confirm that it’s important to carefully monitor help-seeking-behavior in young patients typically manifested in their early 20, especially in terms of psychosis prevention [44].

As for care continuity in the FEP prodromal phase, we notably found that about 84% of our participants with prior contact with mental health centers (both private and public) definitively ended their previous retention in care, presenting to the Pr-EP program as a new specialist access (within the public National Health Service). Only 16.2% of FEP patients were directly referred to our EIP protocol by other mental healthcare services within a generalist-to-specialized care program transition. These results are not surprising considering the low rate of service engagement as care continuity within CAMHS-to-AMHS transition reported in young populations with severe mental illness [45]. In this respect, previous studies examining care continuity in clinical samples with mental disorder also showed that young people referred for treatment from CAMHS to AMHS are more likely to receive a discharge diagnosis of schizophrenia spectrum disorders [46], while hyperkinetic and pervasive developmental disorders decreased the likelihood of transition [47]. In this investigation, no previous diagnosis of psychotic disorder was made.

In this respect, the main psychiatric antecedents before entering the Pr-EP program in the FEP + subgroup were personality disorders (32.8%), anxious-depressive disorder (28.9%), conduct disorder (16.2%), and learning disorder (9.8%) (the last two specifically formulated within past contact with CAMHS). Our rates of past anxious-depressive disorder and past conduct disorder are substantially in line with what was reported in other comparable, international FEP epidemiological studies [18–21]. Differently, Ortiz-Orendain and colleagues [18] reported that previous neuro-developmental disorders (apart from autism) were prevalent (31.3%) in their FEP population. However, in this examination we investigated each neurodevelopmental disorder separately and observed a 9.8% baseline prevalence rate of learning disorder and only 2 participants with past comorbid hyperkinetic developmental disorder together with conduct disorder.

As for comparisons on diagnostic prevalence of psychiatric antecedent at presentation compared to previous research, the most relevant differences interested the presence of a personality disorders and a substance use disorder. Indeed, while personality disorders were the most prevalent psychiatric antecedents in our FEP population, both Ortiz-Orendain and co-workers [18] and Rietdijk and colleagues [21] reported lower baseline prevalence rates. Differently, in the epidemiological research by Rietdijk and co-workers [21], the prevalence rate of substance use disorder at entry (27.1%) was much higher than that reported in our study. In this respect, we hypothesized a frequent failure in Italian young FEP participants to honestly admit substance misuse. As for sociodemographic and clinical features, the findings of this investigation showed that FEP + patients were more likely to be single and to have a longer DUP compared to FEP- ones. This suggests more difficulties in creating stable affective relationships, as well as in maintaining valid support systems. In this respect, it has been reported that FEP patients with effective support systems assisting them in their help-seeking behavior reach mental healthcare services faster, have shorter DUP, and show better treatment engagement [48]. Indeed, social support in FEP individuals (especially, to have a supportive family) notoriously has a positive effect on treatment adherence (although this effect does not seem to persist over time) [49].

Clinical results at presentation also showed that a diagnosis of schizophrenia was more common in the FEP + subgroup, while being referred to the Pr-EP program by emergency room, and having a benzodiazepine prescription were more frequent in FEP- participants.

In this respect, our findings suggest that the absence of previous specialist contact with mental healthcare services is associated to more urgent (“first”) access for psychosis psychopathology in emergency room (especially within a first episode schizophrenia) and a more frequent use of benzodiazepine drug. On the contrary, having had a past contact with psychiatric services could “paradoxically” become a clinical barrier to access EIP programs early and to start timely specialized intervention. The reasons of this finding are not clear and should be carefully explored. However, this may partly explain the reported longer DUP in our FEP + subgroup. Furthermore, in our previous paper, we reported that FEP patients who were referred by emergency room also showed a higher baseline prevalence rate of current suicidal ideation compared to those FEP participants entering the Pr-EP protocol through other sources of referrals [50], confirming a more severe clinical presentation.

Finally, our results also showed an interesting statistical trend in terms of higher HoNOS total scores and higher HoNOS “Impairment” and “Psychiatric Symptoms” domain subscores in FEP + patients compared to FEP- peers across the 2 years of follow-up. These findings overall suggest that FEP individuals with previous contact with mental healthcare services are more likely to manifest severe, prolonged, and compromising psychopathology, maybe as consequence of a diagnosis of schizophrenia or a less effective response to our specialized EIP treatment. Differently, FEP individuals without any prior contact with psychiatric services seem to present with a more striking and acute clinical picture, most commonly leading to emergency room visits. This further supports the well-known evidence that sudden onset of psychosis would not need previous contact, and often would course better [38, 51]. Furthermore, these results lead us to consider psychiatric antecedents in FEP patients as predictors of worst outcome and treatment response over time. Future studies specifically examining the link between personality disorders and substance misuse in subjects with first episode psychosis will be able to clarify the role of self-medication [52].

### Limitations

This study has noteworthy limitations. First, information on psychiatric antecedents was collected retrospectively, which means that the data was registered from medical records. This methodology is prone to information bias, as the data in the medical records may not be accurate or complete.

Second, although representing a major traumatic and stressful event, we did not evaluate the impact of the COVID-19 pandemic on access to our mental healthcare services in the period 2020–2021. Indeed, the restrictions related to the pandemic could potentially affect the referral process to the Pr-EP program. However, we overall found a regular decreasing trend in referral rates over time starting from 2016 (see also the Supplementary Materials [Table S2] for details) and the treated incidence of FEP patients during the COVID-19 pandemic was substantially in line with those reported in 2018 and 2019.

Authors should consider to run again their analyses, by excluding data collected during the COVID pandemic, which should be handled as a separate -independent sample. Furthermore, it should be interesting to compare data collected during the pandemic with those collected in the different time-point.

Therefore, future studies on this topic are needed.

Another limitation is the fact that there was no knowledge about potential previous primary care treatments. Indeed, some patients could have received pharmacological treatment for depressive and anxious symptoms from their general practicioner [53]. This may also have resulted in an underestimation of FEP participants with mental health antecedents.

Fourth, our research was limited to a 2-year follow-up period. Therefore, our findings are comparable exclusively with investigations having longitudinally similar designs. Moreover, this could lead to exclusion of a subpopulation with characteristics that further delay the diagnosis, which may show lower help-seeking behaviors and may suffer greater damage from prolonged DUP. Future studies with longer follow-up duration are thus needed.

Finally, our study was conducted entirely in Italy. Therefore, the results could represent outcomes related to the organization of Italian public mental health services, together with the natural history of psychosis. An international multicenter investigation is thus needed to clarify whether and how different health care organizations may lead to different trajectories of pre-psychotic symptoms.

## Conclusion

The results of this research showed that 41.7% of FEP participants had prior contact with mental healthcare services, and 15% had previous contact with CAMHSs. In 83% of these cases, we documented a care discontinuity. Therefore, greater effort should be made to detect psychiatric antecedents of psychosis in both YMH services and primary care, as well as to favor care continuity during child–adult transition and to better manage the interfaces among specialist services (also in order to reduce the DUP). Indeed, FEP people with previous contact tend to have poorer clinical and functioning outcomes. This leads us to hypothesize previous specialist contact as a predictor of worst prognosis and poorer treatment response over time.

## Supplementary Information

Below is the link to the electronic supplementary material.

Supplementary file1 (DOCX 63 KB)

## Data Availability

The data that support the findings of this research are available on reasonable request from the corresponding author. The data are not publicly available due to privacy and/or ethical restrictions.
