# Supplementary material for: Psychiatric antecedents in young patients with first episode psychosis: what relevance for clinical outcomes?
Source: Eur Arch Psychiatry Clin Neurosci. 2025 Feb 28;275(8):2535–46. doi: 10.1007/s00406-025-01981-6 (PMC12638415; doi:10.1007/s00406-025-01981-6)
Supplement: Supplementary file 1 — Supplementary file1 (DOCX 63 KB) [file 406_2025_1981_MOESM1_ESM.docx]

Table S1 – Kaplan-Meier survival analysis results: comparison on 2-year outcome incidence rates between the two FEP subgroups.

| FEP  subgroup | | 1-cumulative proportion surviving at the time | | | Mean (in months) for 2-year service disengagement rate | | | | | | | |
| --- | --- | --- | --- | --- | --- | --- | --- | --- | --- | --- | --- | --- |
|  |  | Estimate | | SE | Estimate | | | SE | 95% CI | | | |
|  |  |  |  |  |  |  |  |  | Lower bound | | Upper bound | |
| FEP+  FEP-  (Overall) | | .230  .281  - | | .029  .027  - | 20.990  20.007  20.417 | | | .408  .414  .296 | 20.191  19.195  19.837 | | 21.790  20.819  20.998 | |
| 2-year Log Rank (Mantel-Cox) | | | | | Χ^2^ | | | df | p | | | |
|  |  |  |  |  | 1.823 | | | 1 | .177 | | | |
| FEP  subgroup | | 1-cumulative proportion surviving at the time | | | Mean (in months) for 2-year new hospitalization rate | | | | | | | |
|  |  | Estimate | | SE | Estimate | | | SE | 95% CI | | | |
|  |  |  |  |  |  |  |  |  | Lower bound | | Upper bound | |
| FEP+  FEP-  (Overall) | | .292  .265  - | | .034  .029  - | 21.497  21.581  21.545 | | | .360  .308  .233 | 20.792  20.977  21.088 | | 22.203  22.185  22.002 | |
| 2-year Log Rank (Mantel-Cox) | | | | | Χ^2^ | | | df | p | | | |
|  |  |  |  |  | .282 | | | 1 | .595 | | | |
| FEP  subgroup | 1-cumulative proportion surviving at the time | | | | | Mean (in months) for 2-year attempted suicide rate | | | | | | |
|  | Estimate | | SE | | | Estimate | SE | | | 95% CI | | |
|  |  |  |  |  |  |  |  |  |  | Lower bound | | Upper bound |
| FEP+  FEP-  (Overall) | .065  .038  - | | .018  .012  - | | | 23.294  23.661  23.503 | .216  .134  .117 | | | 22.871  23.399  23.273 | | 23.717  23.924  23.734 |
| 2-year Log Rank (Mantel-Cox) | | | | | | Χ^2^ | df | | | p | | |
|  |  |  |  |  |  | 1.801 | 1 | | | .180 | | |

Note. FEP = First Episode Psychosis; FEP+ = FEP participants with past specialist contact; FEP- = FEP participants without past specialist contact; SE = Standard Error; 95% CI = 95% Confidence Intervals; Log Rank = Logarithm Rank Test; = Chi-Squared test; df = degrees of freedom; p = statistical value.

Figure S1 – Kaplan-Meier survival analysis results: survival functions on 2-year outcome incidence rates between the two FEP subgroups across the follow-up.


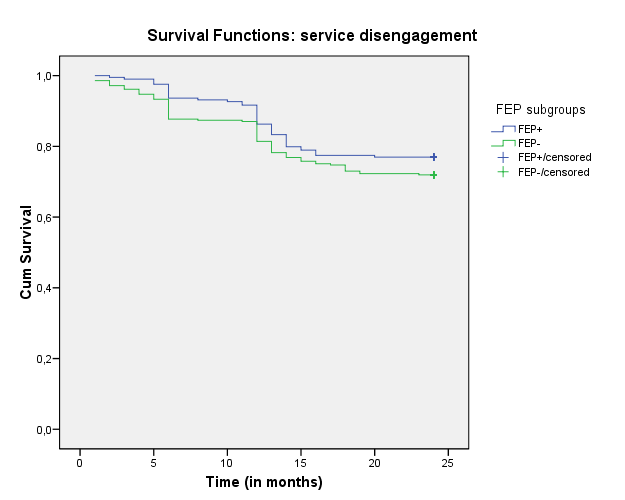


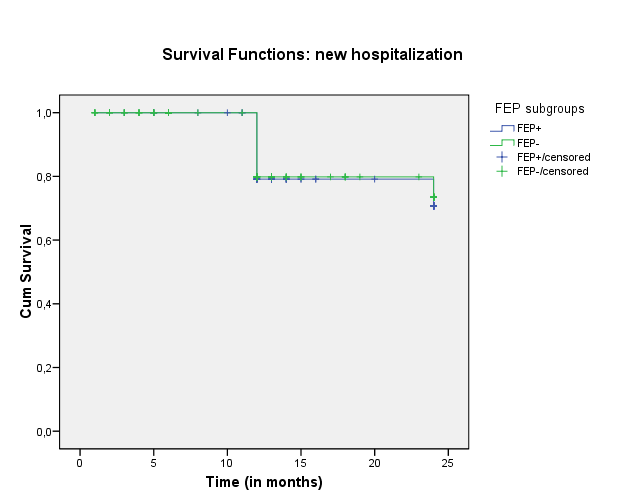


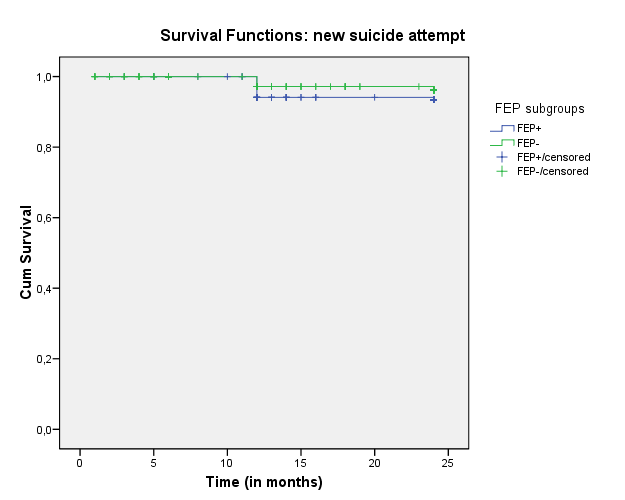


Note. FEP = First Episode Psychosis; FEP+ = FEP participants with past specialist contact; FEP- = FEP participants without past specialist contact.

Table S2 – Treated incidence of FEP patients referred to the Pr-EP program across the recruitment period (2013-2021) (n = 489).


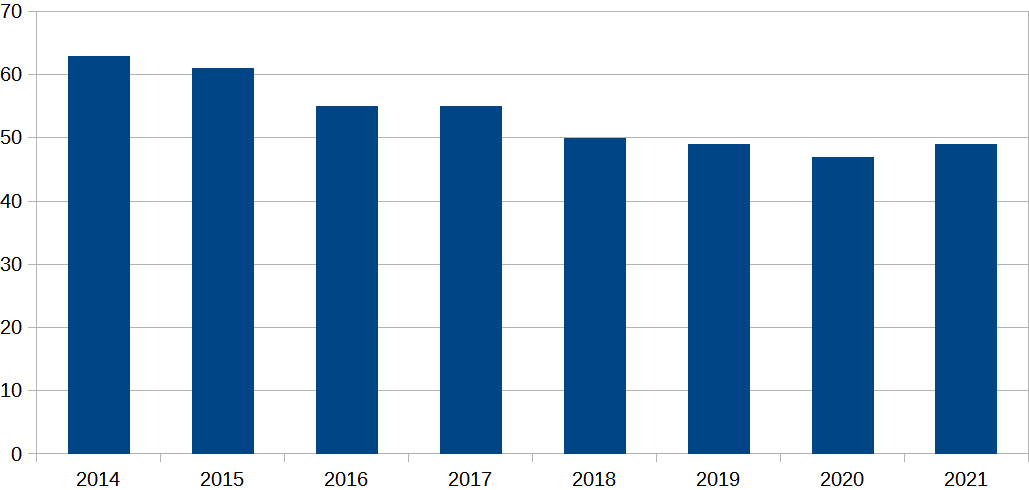
Note - FEP = First Episode Psychosis; Pr-EP = Parma-Early Psychosis.
